# Experimental and bioinformatic analysis of cultured Bovine Endometrial Cells (BEND) responding to interferon tau (IFNT)

**DOI:** 10.1186/s12958-016-0156-y

**Published:** 2016-04-18

**Authors:** Sergio E. Palma-Vera, Ralf Einspanier

**Affiliations:** Institute of Veterinary Biochemistry, Freie Universität Berlin, Oertzenweg 19b, 14163 Berlin, Germany; Leibniz Institute for Farm Animal Biology (FBN), Wilhelm-Stahl-Allee 2, 18196 Dummerstorf, Germany

**Keywords:** Endometrium, Interferon tau, MiRNA, Promoter region

## Abstract

**Background:**

In ruminants, embryo implantation depends on progesterone (P4) and interferon tau (IFNT) controlling endometrial function. IFNT antagonizes bovine endometrial cells (BEND) response to phorbol 12,13-dibutyrate (PDBU) through posttranscriptional regulation of gene expression. We have previously described microRNAs (miRNAs) profiles in bovine endometrium, detecting miR-106a, relevant for embryo maternal communication. In this study, we investigated the expression miR-106a and genes for prostaglandin-endoperoxide synthase 2 (PTGS2), phospholipase A2, group IVA (PLA2G4A), estrogen receptor 1 (ESR1) and progesterone receptor (PR) in response to IFNT in BEND cells and searched for interferon responsive factors (IRFs) binding sites in their promoter genomic regions. The aim of this study was to unravel the molecular mechanisms involved in IFNT signalling and its regulation of miR-106a.

**Findings:**

*PTGS2* showed increased expression under PDBU, which was antagonized by IFNT. IFNT induced expression of *PR* and miR-106a and downregulation of *ESR1* and *PR*. Bioinformatic analyses detected that *PLA2G4A* was associated to IRF-1 and IRF-6, while *ESR1*, *PR* and *PTGS2* were associated to only IRF-6. All genes exhibit one motif per IRF, except miR-106a that had three binding sites for IRF-6.

**Conclusions:**

We report the IFNT regulatory effect on miR-106a expression through IRF-6 in bovine endometrial cells. We identified a set of potential binding sites for IRF-1 and IRF-6 within the bovine genome. A set of candidate gene regions could be characterized where IFNT can act via IRFs to regulate the expression of proteins and miRNAs. Future studies will use these data to detect new IFNT regulatory mechanisms in the endometrium.

## Introduction

Failed embryo implantation is one of the main causes of poor reproductive performance in cattle [1]. Implantation in ruminants depends on uterine receptivity derived from ovarian progesterone (P4) and embryonic interferon tau (IFNT) signalling in endometrial cells. Here, both P4 and IFNT are able to regulate the expression of estrogen receptor 1 (ESR1) [2]. Together, they modulate genes involved in endometrial attachment of the trophectoderm and suppress the luteolytic release of prostaglandin F2 alpha (PGF2alpha) by the endometrium [2–4]. The response of endometrial cells to IFNT has been shown to be dependent of IFN regulatory factors (IRFs) [5]. There are nine mammalian IRFs, which share a conserved 115 aminoacid N-terminal DNA binding domain (DBD) that binds to the promoter region of target genes [6].

Bovine endometrial cells (BEND) [7] provide a model to understand prostaglandin (PG) biosynthesis in response to IFNT. Stimulation of PG production in BEND cells leads to an increased expression of the enzymes prostaglandin-endoperoxide synthase 2 (PTGS2) and phospholipase A2, group IVA (PLA2G4A) and production of PGF2alpha, and these responses are diminished by IFNT, through a transcriptional dependent process [8–11].

MicroRNAs (miRNAs) are short non-coding RNA molecules controlling gene expression [12]. Studies in cattle have identified miRNAs within the endometrium regulating subclinical endometritis and fertility [13, 14]. However, studies are missing describing miRNAs involved in embryo maternal communication. The miR-106a is known to have roles embryo-endometrial cross talk [15–19]. We have previously characterized the expression of miRNAs in bovine endometrium across the estrous cycle and detected the expression of miR-106a [20]. In this study, we aimed to assess the effects of IFNT on miR-106a expression and to predict the location of genomic binding sites for interferon responsive factors (IRFs) that can regulate the expression of genes involved in endometrial response to embryo implantation.

## Material and methods

### BEND cell culture

Immortalization of BEND cells has been previously described [7]. They are distributed by the American Type Culture Collection (ATCC, Manassas, USA), whose indications for handling were followed. BEND cells are able to respond to phorbol 12,13-dibutyrate (PDBU), an activator of protein kinase C (PKC) and mitogen-activated protein kinase (MAPK) signalling pathway, increasing the production of prostaglandins. This effect is antagonized by IFNT [8, 10, 11].

### Experimental design

5 × 10^4^ cells per mL medium (40 % Ham's F-12 (Biochrom), 40 % EMEM (ATCC), 200 U insulin/L (Sigma–Aldrich), 50 μg gentamicin (Biochrom), 10 % FBS (Biochrom), 10 % horse serum (ATCC)) were plated into wells of a 12 well plate (Greiner Bio-One) and grown to ~ 90 % confluence at 37 °C and 5 % CO_2_. Cells were washed with D-PBS and equilibrated in serum free medium for 45 min at 37 °C, 5 % CO_2_. Next, cells were cultured for 6 h with the following treatments: vehicle control, PDBU (100 ng/mL, Sigma–Aldrich), IFNT (50 ng/mL, source see below), P4 (10 ng/mL, Sigma–Aldrich), PDBU + IFNT, PDBU + P4, IFNT + P4, PDBU + IFNT + P4. Doses of IFNT and PDBU were applied as described previously [9], while P4 dose was selected according to the luteal phase levels in cattle [21]. Total RNA was extracted following the instructions of the kit’s manufacturer (mirVana™, Life Technologies). The quality and quantity of the resulting RNA was measured by absorbance at 260 nm (NanoDrop 8000, Thermo Scientific). Recombinant ovine IFNT (antiviral activity, 1 × 10^8^ U/mg) was kindly donated by Dr. F.W. Bazer (Texas A&M University, College Station, TX, USA).

### RT-qPCR for miRNAs and mRNAs

Quantitative RT-PCR was performed as described previously [22] and miRNA was quantified implementing the miR-Q method [23]. For protein coding gene transcripts, primers and annealing temperatures are indicated in Table 1. For miRNAs, primers are indicated in Table 2. All oligonucleotides were purchased from Sigma–Aldrich. Expression levels of mRNA and miRNAs were determined in duplicate and relative gene expression was calculated applying the method described by Livak and Schmittgen [24], correcting for PCR efficiency. Four housekeeper genes (*SDHA*, *ACTB*, *GAPDH*, *SUZ12*) were tested for normalization of protein coding gene expression. The two most stable genes were selected by using the GeNorm algorithm [25]. For miRNA normalization, bta-miR-99a-5p was selected as reference, since its expression was not affected by any of the treatments. All amplicons were validated by DNA sequencing at GATC Biotech AG (Konstanz, Germany).

**Table 1:** List of primers used for quantitative RT-PCR amplification

	Forward primer (5’-3’)	Reverse primer (5’-3’)	Product length (bp)	Annealing temperature (°C)	Gene bank accession
PTGS2	CTG AGT ACT TTT GAC TGT GGG AG	CTC TTC CTC CTG TGC CTG AT	359	60	NM_174445
PLA2G4A	AAA TGT CAG CCA CAA CCC TC	ATG GAG GGT GAA AAG CG	229	56	NM_001075864.1
PR	GAG AGCT CAT CAA GGC AAT TGG	CAC CAT CCC TGC CAA TAT CTTG	227	60	NM_001205356.1
ESR1	AGG GAA GCT CCT ATT TGC TCC	CGG TGG ATG TGG TCC TTC TCT	234	58	AY538775
SDHA	GGG AGG ACT TCA AGG AGA GG	CTC CTC AGT AGG AGC GGA TG	219	60	DQ386895.1
SUZ12	TTC GTT GGA CAG GAG AGA CC	GTG CAC CAA GGG CAA TGT AG	286	60	NM_001205587.1
ACTB	CGG TGC CCA TCT ATG AGG	GAT GGT GAT GAC CTG CCC	266	58	AY141970
GAPDH	CCC AGA AGA CTG TGG ATG G	AGT CGC AGG AGA CAA CCT G	306	32	U85042

**Table 2:** Oligonucleotides for miR-Q PCR amplification

	Primer sequence (5’-3’)	
bta-miR-106a	RT6-miRNA	TGT CAG GCA ACC GTA TTC ACC GTG AGT GGT TAC CTG
	miRNA-rev	CGT CAG ATG TCC GAG TAG AGG GGG AAC GGC GAA AAG TGC TTA CAG TG
bta-miR-99a-5p	RT6-miRNA	TGT CAG GCA ACC GTA TTC ACC GTG AGT GGT ACA AGA
	miRNA-rev	CGT CAG ATG TCC GAG TAG AGG GGG AAC GGC G AAC CCG TAG ATC CGA TCT

### Statistical and bioinformatics analysis

Data for gene expression are presented as boxplots. Depending on whether or not data showed normality, analysis of variance (ANOVA) or Kruskal-Wallis rank sum test were applied, followed by the post-hoc tests Bonferroni or Mann-Whitney *U*-test, respectively.

Candidate IRFs binding sites to DNA promoter gene regions were performed in R, applying the corresponding Bioconductor workflow. Binding motifs for IRFs were retrieved from MotifDb and then matched to the promoter regions of protein coding and miRNA coding genes of the bovine genome (UMD3.1.1).

## Results and discussion

### Regulation of PTGS2 and PLA2G4A

**Fig. 1:**
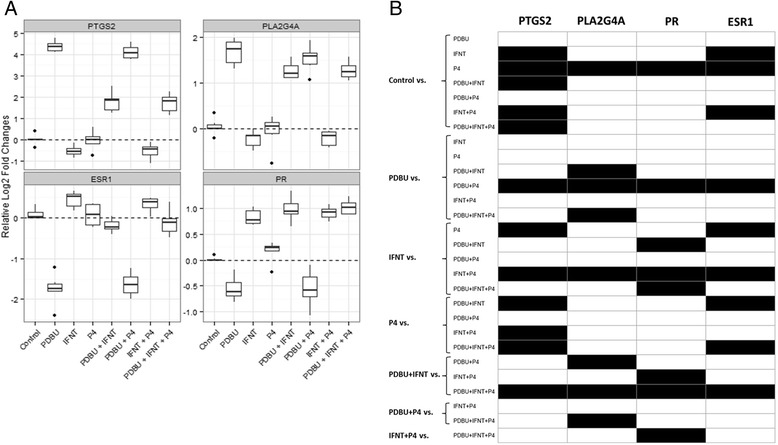
Regulation of PTGS2, PLA2G4A, ESR1 and PR expression in BEND cells. **a** Normalized log 2 fold change mRNA expression of characteristic genes for BEND cells in response to treatment with PDBU, IFNT, P4 and their combinations. Transcript expression was normalized to a combination of two housekeeping genes (SUZ12 and SDHA). **b** Matrix of significances (white: *p* < 0.05; black: *p* > 0.05). For each experiment, six biological replicates were used. Outliers are indicated as single dots above or below the whiskers

Previous studies have described the antagonizing effect of IFNT when PDBU was added to BEND cells. The result was a reduction of the mRNA of *PTGS2* and *PLA2G4A* [8, 9, 11]. In our study, *PTGS2* and *PLA2G4A* were upregulated by PDBU. For *PTGS2*, the PDBU effect was antagonized by IFNT, but this was not observed for *PLA2G4A* (Fig. 1). The lack of *PLA2G4A* regulation implies a stronger effect of IFNT on the expression of *PTGS2* and a reduced effect on the expression of *PLA2G4A*. It has been shown that IFNT antagonizes the effect PDBU on the protein levels of *PLA2G4A* [9]. Thus, it could be possible that at the mRNA level, this effect remains inconspicuous. Nevertheless, the downregulation of *PTGS2* corroborates the validity of our assays.

### IFNT upregulates PR and PDBU downregulates estrogen and progesterone receptors

We detected a significant upregulation of progesterone receptor (PR), but not ESR1 transcripts upon IFNT signalling (Fig. 1). To our knowledge, these results have not been reported in the BEND cell model before. From the physiological point of view, upregulation of *PR* by IFNT is reasonable, due to the positive role of P4 in maintaining pregnancy and its permissive effect on IFNT activity. However, in vivo implantation events are preceded by loss of expression of *PR* and *ESR1* [4]. Such discrepancy might be explained by the nature of the BEND cell line, where not all physiological properties are preserved after establishment.

However, IFNT was able to induce a significant increase of PR mRNA expression. This effect remained when IFNT was combined with P4 and PDBU. On the other hand, *ESR1* and *PR* expression was reduced in response to PDBU and this effect was reversed by IFNT in different magnitudes: *ESR1* returned to basal levels and *PR* was 2 folds upregulated. Unlike IFNT, P4 was not able to reverse the effects of PDBU on *ESR1* and *PR* expression.

### Expression of miR-106a is regulated by IFNT

**Fig. 2:**
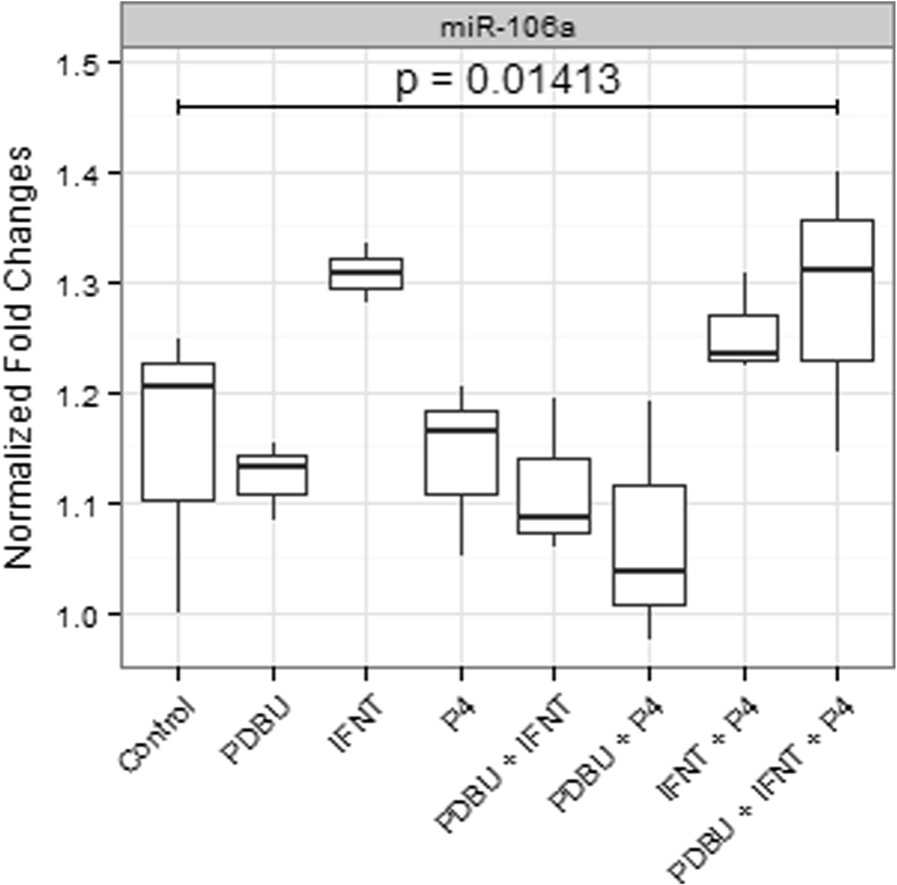
Normalized fold change expression of miR-106a for BEND cells. Expression was normalized to a stable unregulated miRNA (bta-miR-99a-5p). For each experiment, six biological replicates were used

An overall significant effect was detected on the expression of miR-106a (Fig. 2). This effect was most likely due to the activity of IFNT, which increased the expression of miR-106a approximately 30 % when applied alone. Also, when IFNT was applied with P4 and PDBU plus P4, a similar increment was detected. The only treatment group where the regulatory effect of IFNT was not observed when IFNT was added in combination with PDBU. This indicates that PDBU might counter-regulate the activity of IFNT and P4 ameliorates this effect.

Evidence showed that miR-106a responds to IFNT alone and in combination with P4. This is physiologically relevant, since progesterone is permissive for IFNT activity [4]. On the other hand, when IFNT combined with PDBU were applied, miR-106a expression was not affected, pointing towards a counter-regulation of PDBU over IFNT. Considering that PDBU action is analogous to the activity of oxytocin, e.g. induction of PGF2alpha production, this event parallels the physiology of embryo maternal communication. Therefore, it is possible that miR-106a contributes to the control of endometrial responses to IFNT and oxytocin.

### IRF-1 and IRF-6 are found in the promoter regions of regulated genes

**Fig. 3:**
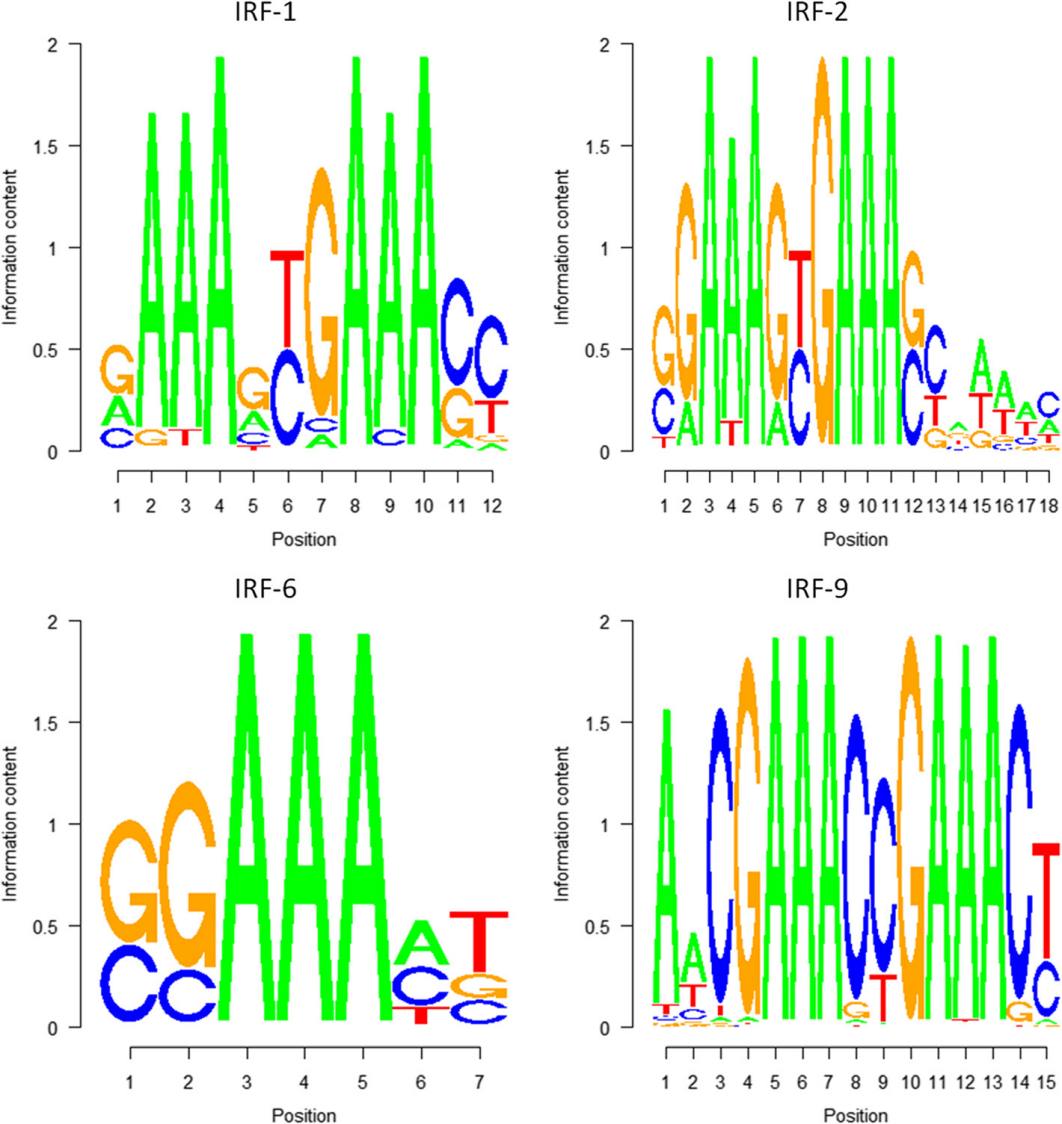
Sequence logos for IRF-1, IRF-2, IRF-6 and IRF-9 binding motifs. Sequences were retrieved from MotifDb and searched in the bovine genomic regions corresponding to the promoters of *PTGS2*, *PLA2G4A*, *ESR1*, *PR* and *MIR106A*

We searched for the binding sites of IRFs in the bovine genome at the promoter site of known genes. Binding sites were determined by the presence of DNA motifs for a specific IRF. These motifs can be visualized as sequence logos in Fig. 3, showing the frequency of nucleotides at each position of the sequence. IRFs binding sites lengths ranged from 7 (IRF-6) to 18 (IRF-2), all having adenines as the most prevalent nucleotides. IRFs were selected based on previous studies, as they are known to be present in the endometrium of ruminants [5]. For protein coding genes, there were severe differences in the number of binding sites: IRF-6 was identified more than 40 thousand times, while IRF-1, 2 and 9 lay far behind (Table 3). A similar pattern was detected for miRNA coding genes. We decided to search for promoter binding sites at genes relevant for BEND function and miR-106a, leaving out thousands of genomic regions where IRFs can bind. These regions may regulate the expression of other genes and miRNAs. Future experimental studies will define what their roles are in order to detect pathways controlled by IFNT in BEND cells.

**Table 3:** Number of binding sites per IRF for protein and miRNA coding genes promoter regions

	IRF-1	IRF-2	IRF-6	IRF-9
Protein coding genes promoters	2603	83	40315	5
miRNA coding genes promoters	54	2	1135	0

We found that for all the protein coding genes relevant to BEND cell function, IRF-1 and 6 had binding sites in the promoter regions. Interestingly, miR-106a was 3x enriched for IRF-6 in its promoter region (Table 4). In this context, it has been reported that IRF6 could play a critical role in endometrial gene expression and trophectoderm growth [5]. This can explain the upregulation of miR-106a when BEND cells are treated with IFNT and imply a potential role of this miRNA in embryo maternal communication in cattle.

**Table 4:** IRF enrichment in promoter regions for *ESR1*, *PR*, *PTGS2*, *PLA2G4A* and *MIR106A*

IRF	Number of binding sites	Gene
IRF-1	1	*PLA2G4A*
IRF-6	1	*PR*
IRF-6	1	*PTGS2*
IRF-6	1	*ESR1*
IRF-6	1	*PLA2G4A*
IRF-6	3	*MIR106A*

## Conclusions

We present evidence that miR-106a in a bovine endometrial cell culture (BEND) is regulated by IFNT. IFNT might induce binding of IRF-6 to the promoter region of miR-106a inducing its expression. This study shows that bioinformatic methods for detecting IRF binding sites in the genome can explain and support the observed experimental data. In the future, these data sets may be used to search for more candidate genes involved in embryo maternal communication. Finally, the BEND cell model, provides a simple and reliable cell system for discovering key regulators of bovine fertility, such as miRNAs.s

## Abbreviations

BEND: bovine endometrial cells
ESR1: estrogen receptor 1
IFNT: interferon tau
IRF: interferon responsive factor
MAPK: mitogen-activated protein kinase
miRNA: MicroRNA
P4: progesterone
PDBU: phorbol 12,13-dibutyrate
PG: prostaglandin
PGF2alpha: Prostaglandin F2 alpha
PKC: protein kinase C
PLA2G4A: phospholipase A2, group IVA
PR: progesterone receptor
PTGS2: prostaglandin-endoperoxide synthase 2
